# Plasma Concentrations of Oseltamivir and Oseltamivir Carboxylate in Critically Ill Children on Extracorporeal Membrane Oxygenation Support

**DOI:** 10.1371/journal.pone.0010938

**Published:** 2010-06-03

**Authors:** Enno D. Wildschut, Matthijs de Hoog, Maurice J. Ahsman, Dick Tibboel, Albert D. M. E. Osterhaus, Pieter L. A. Fraaij

**Affiliations:** 1 Intensive Care and Department of Paediatric Surgery, Erasmus MC - Sophia Children's Hospital, Rotterdam, The Netherlands; 2 Department of Paediatrics, Erasmus MC - Sophia Children's Hospital, Rotterdam, The Netherlands; 3 Clinical Pharmacology Unit, Department of Hospital Pharmacy, Erasmus MC, Rotterdam, The Netherlands; 4 Department of Virology, Erasmus MC, Rotterdam, The Netherlands; Aga Khan University, Pakistan

## Abstract

**Introduction:**

To evaluate the effect of extracorporeal membrane oxygenation (ECMO) support on pharmacokinetics of oseltamivir and oseltamivir carboxylate (OC) in children.

**Methodology:**

Steady state 0–12 hour pharmacokinetic sampling was performed in new influenza A (H1N1) infected children treated with oseltamivir while on ECMO support. Cmax, Cmin and AUC_0–12 h_ were calculated. The age-specific oseltamivir dosage was doubled to counter expected decreased plasma drug concentrations due to increased volume of distribution on ECMO support.

**Principal Findings:**

Three patients were enrolled aged 15, 6 and 14 years in this pharmacokinetic case series. For two children the OC plasma concentrations were higher than those found in children and adults not on ECMO. These increased plasma concentrations related to the increased oseltamivir dosage and decreased kidney function. In one patient suboptimal plasma concentrations coincided with a decreased gastric motility.

**Conclusion:**

Oseltamivir pharmacokinetics do not appear to be significantly influenced by ECMO support. Caution is required in case of nasogastric administration and decreased gastric motility. Due to the limited number of (paediatric) patients available further multicenter studies are warranted.

## Introduction

Currently the first influenza pandemic of this century is almost at its end. The new variant influenza A (H1N1) virus appears to be relatively mild compared to its pandemic predecessors. [1] Still, a life threatening disease pattern not characteristic for seasonal influenza has been identified in often young patients infected with new variant influenza A (H1N1). The clinical picture of this severe illness is one of Acute Respiratory Distress Syndrome (ARDS), sometimes associated with septicaemia-like symptoms. While relatively rare, these cases impose a burden on intensive care units. [2], [3], [4]

The optimal treatment for children and adolescents with influenza associated ARDS has not yet been established. Based on recent data, mostly obtained in adults, the use of extra corporeal membrane oxygenation (ECMO) support in combination with the use of neuraminidase inhibitors appears to be a feasible option. [3] ECMO support is associated with altered pharmacokinetics for several drugs. This is due to the increment of the total circulation volume and adherence to plastic tubing and membranes. [5] Suboptimal plasma concentrations of neuraminidase inhibitors may be associated with reduced antiviral effectiveness of the drug and the development of viral drug resistance. [6] The aim of this study is to evaluate the effect of ECMO support on plasma concentrations of oseltamivir and oseltamivir carboxylate (OC) in children.

## Methods

This is a prospective analysis of pharmacokinetic data from new influenza A (H1N1) infected children (0–18 years) treated with oseltamivir that required ECMO support (Medtronic Sh. 70 USP class VI 3/8×3/32 superTygon®, Medtronic, Minneapolis, USA). As routine protocol the age-specific oseltamivir dosage was doubled to counter expected decreased plasma drug concentrations due to ECMO support. This resulted in the following oseltamivir dosing regimen: <15 kg: 60 mg/day q12 h, 15–23 kg: 90 mg/day q12 h, 23–40 kg: 120 mg/day q12 h and >40 kg: 150 mg/day q12 h. Medication was administered though nasogastric or duodenal tube. According to our hospital based ECMO protocol continuous venovenous hemofiltration (CVVH) (Multiflow 100 Hospal, Lyon, France) was performed during ECMO as a standard treatment.

Twenty-four hours after initiation of ECMO support blood samples were obtained from the ECMO system in BD Hemocard™ EDTA/NaF tubes. Sampling was performed at 0-1-2-4-6-12 hours after oral administration of oseltamivir suspension 15 mg/ml (patient 1) and 12 mg/ml (patient 2 and 3). After sampling and centrifugation, the supernatant serum was stored at −80°C and shipped in batch. Plasma concentrations for oseltamivir and OC were determined by PRA, Bio-analytical Laboratory Assen, the Netherlands by a commercial validated HPLC assy.

Medical data was collected using a Patient Data Management System. Written informed consent was obtained from parent or care takers prior to enrolment. The study was approved by the institutional medical ethics committee (Medisch Ethische Toetsings Commissie Erasmus MC (METC). METC#-2006-355 and ABR#NL14729.078.06.)

## Results

Three patients were enrolled (1 girl, 2 boys) aged 6, 14 and 15 years in this pharmacokinetic case series. A total of 17 samples (6, 6 and 5 samples each) were available for analysis. None of the patients had a medical history that could influence the oseltamivir pharmacokinetics. All patients required ECMO due to ARDS. Patient 1 and 2 received enteral feeding and tamiflu suspension via a duodenal tube. Patient 3 had severe gastro-enteric bleeding and decreased gastric motility with gastric residue as a result of septicaemia accompanied with diffuse intravascular coagulopathy. Medication in this patient was administered via a gastric tube. Patient 1 and 3 had decreased renal function expressed by increased creatinine concentrations at the time of sampling (see table 1). ECMO flow rates and hemofiltration rates were not adjusted during sampling.

**Table 1:** Baseline characteristics of patients.

Patient	1	2	3
Age (years)	15	6	14
Dosage (Q12 h)	150	120	150
Dosage (Q12 h/kg)	3	4	2,7#
Sex	Female	Male	Male
Creatinine (µmol/l)	88	32	100
Formulation and route of administration	Suspension, Duodenal tube	Suspension, Duodenal tube	Suspension, Gastric tube
**Oseltamivir**			
Cmax (ng/ml)	92.4	41.4	3.4
Cmin (ng/ml)	1.9	0	0
AUC_0–12 h_ (ngxh/ml)	232.9	87.4	25
**Oseltamivir carboxylase**			
Cmax (ng/ml)	1300	548	224
Cmin (ng/ml)	736	236	77,2
AUC_0–12 h_ (µgxh/ml)	10642	3211	978,1

# Weight estimated, due to critical illness and later death impossible to weigh.

**Figure 1:**
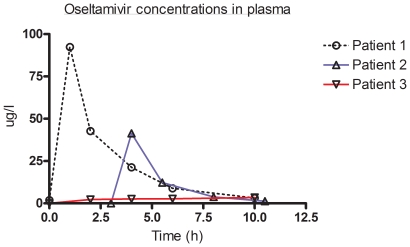
Oseltamivir concentrations in plasma. Plasma concentrations (µg/l) are depicted for each individual patient in time (h). Individual patients are marked with a colour code and maker: patient 1: 0, black; patient 2 ▵, blue and patient 3: ▿,red.

**Figure 2:**
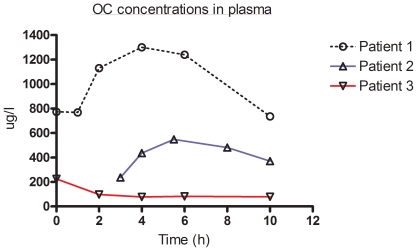
OC concentrations in plasma. Plasma concentrations (µg/l) are depicted for each individual patient in time (h). Individual patients are marked with a colour code and maker: patient 1: 0, black; patient 2 ▵, blue and patient 3: ▿,red**.**

The results of the pharmacokinetics concentrations of oseltamivir and OC are presented in table 1 and figures 1 and 2. In patient 3 suboptimal plasma concentrations were observed for both the parent drug and OC. These coincided with a decreased gastric mobility and nasogastric medication administration. For none of the patients adverse medication reactions were reported

## Discussion

In this pharmacokinetic case study high plasma concentrations for OC were achieved in two out of three patients. Both patients had plasma concentrations that were almost two fold higher compared to historical controls in children aged 3–5 years and 13–18 receiving 2 mg/kg oseltamivir. [7], [8] The elevated plasma concentrations found in our study reflect in part the higher dosing used in our patients. In addition, the (mild) renal impairment seen in patient 1 may also have led to an increase in plasma OC concentrations. In a study by He et al. this has also been shown in adults with mild to severe renal failure. [9]

The plasma concentrations found in this case series show a marked variance. This was previously also seen in non critically ill children. [7], [8] Age related changes in the clearance of OC may be an additional explanation. [7] Patient 3 clearly had suboptimal serum concentrations of both oseltamivir and OC. In this patient the absorption of oseltamivir was severely impaired due to gastric bleeding and decreased gastric motility. In critically ill adults two studies report that oseltamivir can be safely used and is adequately absorbed following nasogastric administration. [10], [11] Our finding warrants caution in patients with severe GI problems, not only in ECMO patients but in all critically ill patients with GI problems. We propose that in these patients, conversion to inhaled or when available intravenous medication (i.e. zanamivir) is indicated.

Due to the limited sample size definite dose recommendations can not be made based on our findings. Still this is the first study to show that adequate plasma concentration of oseltamivir and oseltamivir carboxylate can be achieved in critically ill patients on ECMO. The two fold dose increment that we did in this study does not seem to be necessary to achieve adequate plasma concentrations. However, further research is needed to confirm these findings. A multicenter approach would be preferable, to allow for sufficient inclusions in an acceptable time period.

In conclusion oseltamivir pharmacokinetics do not seem to be significantly influenced by ECMO support. Caution is required in case of nasogastric administration and decreased gastric mobility. In these patients another route of antiviral medication should be considered. We propose to conduct further study on this subject in a lager group of patients
